# Introducing personalized patient care in overactive bladder management using the MedRing OAB system for intravaginal oxybutynin administration

**DOI:** 10.1080/10717544.2026.2617683

**Published:** 2026-01-24

**Authors:** S. I. Peltenburg, I. Koopmans, O. Heerema-Snoep, E. S. Klaassen, M. J. Juachon, A. Otten, N. B. Klarenbeek

**Affiliations:** a Center for Human Drug Research, Leiden, The Netherlands; b Leiden University Medical Centre, Leiden, The Netherlands; c Product Usability Department, LiGalli BV, Leiden, The Netherlands

**Keywords:** Overactive bladder, OAB, MedRing, oxybutynin, intravaginal, drug delivery device, personalized care, smartphone operated, bluetooth, FemTech

## Abstract

Although numerous drugs have been developed for intravaginal administration, the implementation of personalized intravaginal treatment options is limited. The MedRing overactive bladder (OAB) system is a medical device for intravaginal oxybutynin administration via patient-controlled schedules. The primary aim was to assess the feasibility, tolerability, and safety of intravaginal oxybutynin administration via the MedRing OAB system. Second, the functioning of the MedRing OAB system, user satisfaction and quality of life (QoL) were assessed. Female OAB patients were included to receive the MedRing OAB system. Treatment was divided into three periods with increasing dosing flexibility: 2 mg at three fixed timepoints daily, 2 mg at three patient-defined timepoints daily, and flexible dosing up to 6 mg/day of 1 or 2 mg doses. Feasibility, tolerability, satisfaction, and QoL were assessed via questionnaires, safety via treatment-emergent adverse events (TEAEs), device deficiencies (DDs) and physical examination and functioning via pharmacokinetics and MedRing logs. Thirteen patients were enrolled, of whom three patients discontinued the study prematurely. Most patients reported low user burden, found the system practical and expressed positive opinions. The TEAEs were consistent with known oxybutynin effects and local TEAEs were comparable to other intravaginal devices. Most DDs were synchronization difficulties, which improved after a software update. After 10 minutes, oxybutynin levels were detected in 12 of the 13 patients. This study showed that the MedRing OAB system appears to be a feasible, tolerable and safe alternative intravaginal oxybutynin administration for 28 days in OAB patients, offering a potential alternative to existing treatment options and introducing personalized patient care.

## Introduction

Although numerous drugs have been developed for the intravaginal route, this delivery mode has limited personalized patient care options. Intravaginal rings like hormonal contraceptive and HIV prevention rings as well as hormonal suppletion or antibiotic ovules are primarily developed as slow-release drugs (Nel et al. 2016). These continuous intravaginal therapies either lack immediate-release treatment options or require repeated insertions, which hinder truly personalized patient care.

**Figure 1.**
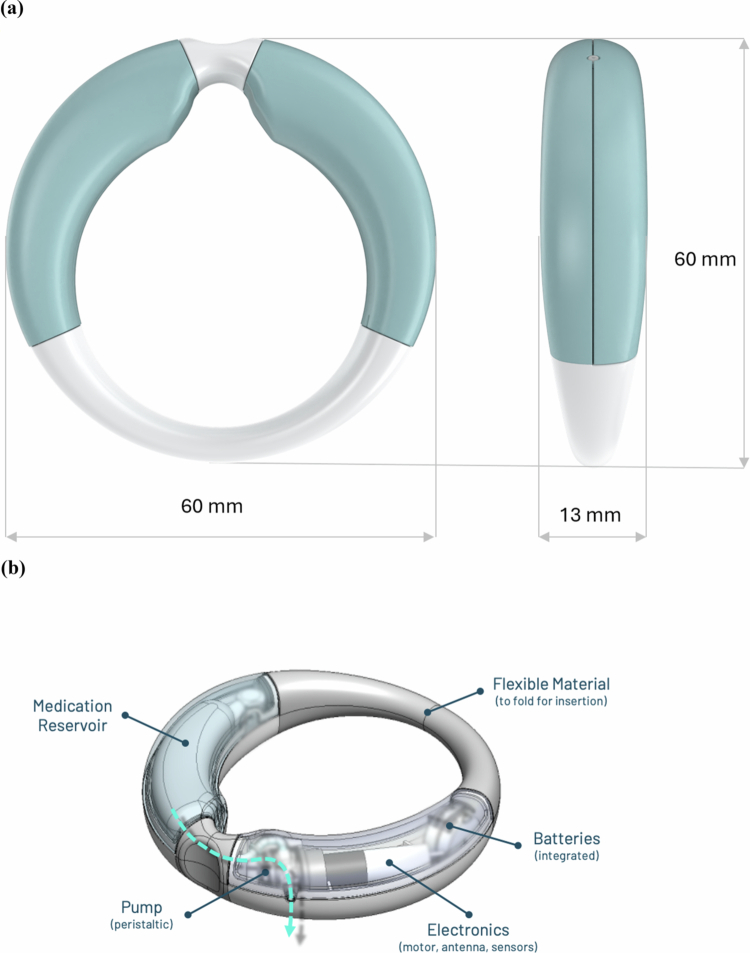
The MedRing OAB. (a) The dimensions of the MedRing OAB. (b) The Alpha 2.1 version, including the different parts of the MedRing. The arrow depicts the route of oxybutynin from the reservoir to the outlet.

The MedRing is a class IIb medical device developed for intravaginal drug administration. The MedRing overactive bladder (OAB) system consists of a ring for intravaginal use, the MedRing OAB, which is controlled by a smartphone application (Figure 1). Via the smartphone application, patients can set up and adjust treatment schedules without the need for removal of the device. Patients can set up a symptom-based treatment schedule tailored to their individual needs including nightly preprogrammed drug administration. Additional functions such as ‘give next dose now’ allow self-initiated drug administrations within safety limits. These options combined enable automated drug delivery to advance personalized patient care.

OAB patients could potentially benefit from the MedRing OAB system. They experience frequent, urgent and nightly micturition, which has a substantial impact on their quality of life (QoL) (Bartoli et al. 2010), and require frequent oral drug administration with oxybutynin, the standard treatment. Current oxybutynin treatment options include immediate release oral tablets three times a day (t.i.d.) or slow-release transdermal patches. These treatment options have limitations due to a high incidence of side effects, the need for frequent dosing, or the absence of an immediate effect (Douchamps et al. 1988; Baldwin and Keating 2009; Jirschele and Sand 2013; Cohn et al., 2016). These limitations lead to discontinuation rates between 33% and 71% (Gopal et al. 2008; Geoffrion et al. 2012). The MedRing OAB system could combine the benefits of both reduction of side effects and allowing convenient and discrete personalized treatment.

The MedRing has previously been tested for oxybutynin administration, which showed adequate intravaginal absorption (de Laat et al. 2023). A more recent study showed that the MedRing filled with oxybutynin (MedRing OAB) resulted in a reduced incidence and severity of the anticholinergic side effect dry mouth, attributed to lower *N*-desethyloxybutynin metabolite (DEOB) concentrations resulting from bypassing the hepatic first-pass effect. Moreover, the MedRing OAB was well tolerated and safe over a period of 72 hours (Peltenburg et al. 2025, submitted). This previous study was performed over a relatively short period and did not assess user experiences. Therefore, the present study was designed to systematically evaluate the user experience of the MedRing OAB system over its intended 28-day use period in an outpatient setting.

The primary aim of the present study was to assess the feasibility, tolerability and safety of intravaginal oxybutynin administration in OAB patients via the MedRing OAB system for 28 days in an outpatient setting. Second, the functioning of the MedRing OAB system, user satisfaction and quality of life of OAB patients were assessed.

## Materials and methods

### Patients

Female patients, aged 18–80 years and diagnosed with an OAB confirmed by a physician were eligible for inclusion in the study if they were in generally good health. OAB was defined as urinary urgency, usually with urinary frequency, and nocturia, with or without urgency urinary incontinence' according to the guidelines of the International Continence Society and International Urogynecological Association (Haylen et al. 2010). Patients were excluded if they presented clinically significant abnormalities on 12-lead electrocardiograms (ECGs), in laboratory test results, or during gynecological inspection or in Pap smear findings, if they had received study-specific prohibited drug, if they had no history of vaginal intercourse or if they had a pessary or a clinically relevant genital or vaginal prolapse of POP-Q stage 2 or higher.

Patients were recruited via media advertisements and invited for medical screening. Prior to any study-related activities, all patients were required to sign a written informed consent. A minimum wash-out period of five times the half-life was employed for current OAB medication. Sample size was not determined with a power calculation, because of the exploratory nature of this study. Instead, to address the study objectives, it was estimated that 12 patients would provide sufficient information. For a better generalizability, both premenopausal and postmenopausal women were included with at least 4 in each group.

### Material and methods

#### Study design

This study was a single-center, longitudinal, exploratory, open-label study to assess the feasibility, safety, and tolerability of intravaginal administration of oxybutynin via the MedRing OAB system for 28 days. The study was conducted at the Center for Human Drug Research (CHDR) in Leiden, the Netherlands.

After confirmed eligibility, patients received their MedRing that was paired with the patient's smartphone application via a Bluetooth connection. The study physician primed the MedRing OAB using the smartphone application to remove residual air from the outlet and reservoir. After gynecological inspection, patients were subsequently instructed on the insertion and removal of the MedRing and how to synchronize the smartphone application. Time of the first dose was set by the physician via the patients' smartphone application and administered at the site, after which patients were discharged. All patients were instructed to keep their usual genital hygiene habits unchanged, and to remove the MedRing only in circumstances they regarded necessary. During the next 28 days, patients visited the site at days 7, 14, 21, and 28. At day 28, the MedRing was removed and the smartphone application was uninstalled. A follow-up visit was planned 7 days later.

**Figure 2.**
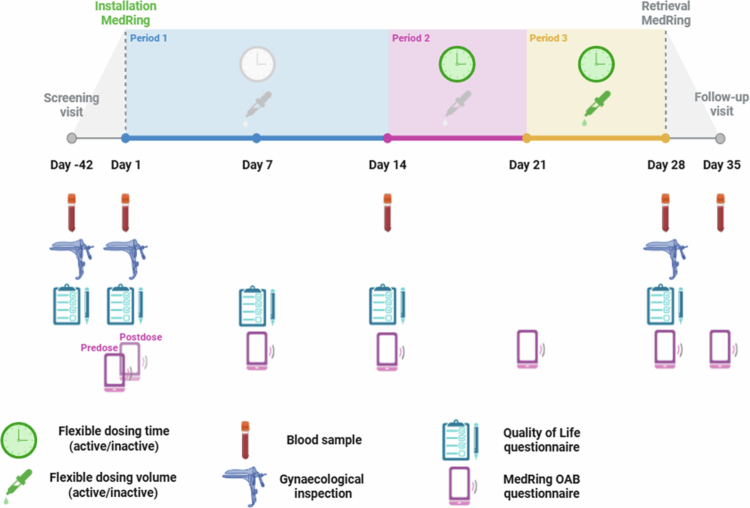
Study design. Abbreviations: OAB = overactive bladder.

The study consisted of three consecutive treatment periods (Figure 2). In the first treatment period, patients received 2 mg oxybutynin three times a day (t.i.d.) at 0:00, 8:00 and 16:00 hours for 14 days. In the second treatment period, patients were instructed to administer 2 mg t.i.d. at self-selected timepoints for 7 days, including moving a dose forward with the function ‘give next dose now.’ Finally, in the third treatment period, patients were instructed to dose ‘as needed’ with 1 or 2 mg oxybutynin doses with a maximum dose of 6 mg in 24 hours for 7 days. For all periods, a minimum dosing interval of 3 hours was required. Each time a new period was initiated and more flexibility was allowed, patients were instructed to adjust their schedule to the most optimal schedule for relief of OAB symptoms.

Interruption or premature discontinuation of the study drug could be triggered due to an adverse event, a diagnostic or therapeutic procedure, an abnormal assessment, or for administrative reasons, in particular, withdrawal of the patient's consent.

#### Study treatment and dosing rationale

All patients received intravaginal oxybutynin treatment via the MedRing OAB system. The MedRing OAB system consisted of the MedRing OAB, a smartphone application and a web application. The MedRing (version Alpha 2.1) was filled with oxybutynin hydrochloride 100 mg/mL. The dose of 2 mg t.i.d. was selected based on historical data of the oxybutynin plasma levels (area under the curve (AUC)) after oral and transdermal administration and the interim results of the previous MedRing study (Peltenburg et al. 2025, submitted) (Douchamps et al. 1988; Zobrist et al. 2003; Appell et al. 2003; Baldwin and Keating 2009; de Laat et al. 2023).

#### Feasibility

Feasibility of the MedRing OAB system was assessed at day 28 with a questionnaire specifically developed for the present study: the MedRing OAB questionnaire. The questionnaire was electronically answered via a third application, the ePRO application. The ePRO application was able to send notifications and reminders (Coons et al. 2009). In the event of a missed questionnaire, the physician assessed whether reliable data could still be obtained during the next visit, and missing data were collected on a paper form if the patient could recall the answers. Patients completed the questionnaire in Dutch. A translation of the questionnaire in English can be found in Supplementary Questionnaire 1.

**Figure 3.**
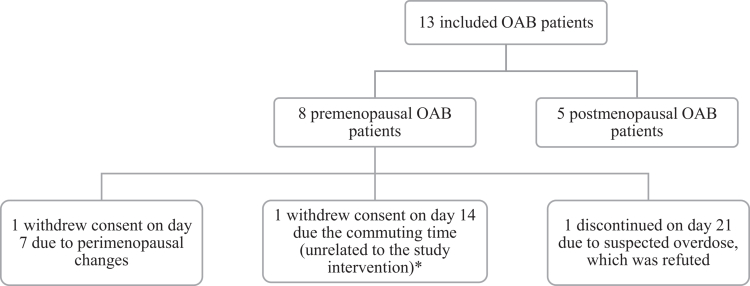
Patient disposition. Abbreviations: OAB = overactive bladder. *This premenopausal patient was replaced by a postmenopausal patient.

The questionnaire consisted of 15 questions covering physical and emotional burden of the use of the MedRing OAB system, 7 questions regarding its practical use, 6 questions regarding the ‘as needed’ dosing, and 11 questions related to the general opinion of the system. Answers were given by setting a slider on a visual analog scale (VAS) ranging from 0 to 10, with a default position of 5, with 0 indicating a negative or dissenting response, and 10 indicating a positive or affirming response. The outcome of the four primary outcome questions regarding menstruation, sexual activities, work and work-related activities, and physical activities varied among the participants, which is why these questions were assessed conditionally: patients first answered if the question was applicable to them. If applicable, a VAS slider appeared to rate the experienced burden; if not, the VAS slider was skipped (Figure 3, questions marked with an asterisk).

#### Safety and tolerability

Safety and tolerability endpoints included safety parameters, findings during gynecological inspection and treatment-emergent adverse events (TEAEs).

Adverse events (AEs) were collected electronically via the ePRO application and during clinical study visits. In the ePRO application, specific AEs such as dry mouth, dry eyes, obstipation, less sweating, and dry skin were scored on a VAS scale (VAS) (0–10), and an open field was used to report other TEAEs. During a clinical visit, TEAEs and data regarding concomitant medication use were collected and reviewed for their relationship with the use of oxybutynin and the device. Gynecological inspection was conducted before insertion, after retrieval of the MedRing and when considered necessary in cases of symptoms or a TEAE (Figure 2). Vital signs were measured during each study visit. At days 1, 14, and 28, safety laboratory measurements were performed (Figure 2).

#### Functioning of the MedRing

The performance of the MedRing OAB system was evaluated for 28 days via device deficiencies (DDs), the MedRing logs in the web application, and postcalibration output tests.

DDs were defined as any inadequacy in quality, durability, reliability, safety, or performance of the MedRing OAB system and categorized into three types: 1) a DD of the MedRing OAB that would have a direct effect on the functioning of the device; 2) a DD in the smartphone application that would result in incorrect data logging or suboptimal performance of the MedRing OAB; and 3) a DD in the MedRing logs potentially resulting in inaccurate display of the data.

Through the web application, the physician could access the uploaded data of the MedRings and the smartphone application in the study. The following data were stored: intravaginal temperature assessments at 10-minute intervals, synchronization attempts between the MedRing and the smartphone application, dosing time and volume, the number of times the smartphone application was opened, schedule adjustments in the smartphone application and openings of the instructions and frequently asked questions in the smartphone application.

After retrieval at day 28, the dosing accuracy of all the MedRings was checked with a postcalibration output test. For this test, a MedRing was programmed for a 2 mg dose, after which the volume of this dose was recorded. A measured volume within a 20% margin of a 2 mg dose was considered successful.

#### Pharmacokinetics

Blood samples were obtained at days 1, 14, and 28 to measure oxybutynin and DEOB plasma levels using a validated liquid chromatography-tandem mass spectrometry method with an LLOQ of 0.100 ng/mL. At day 1, samples were taken at 5, 10, and 60 minutes after the first 2 mg dose. At days 14 and 28, samples were taken 60 minutes after a 2 mg dose.

#### Satisfaction and quality of life

At days 1, 7, 14, 21, 28, and 35, the MedRing OAB questionnaire was taken to measure satisfaction (Figure 2). Moreover, to measure the QoL, a validated, short-form QoL questionnaire was taken at day 1 predose, 7, 14, and 28 to assess the QoL between visits (Figure 2) (Groenendijk et al. 2019).

#### Preliminary clinical effect

An exploratory analysis of the void frequency and volume was performed on the basis of the data recorded during the toilet visits. Patients were instructed to register each void manually in the smartphone application during the third treatment period only. Due to the variability and the apparent incomplete registrations, these data are not reported.

#### Statistical analysis

Descriptives of the premenopausal and postmenopausal subjects were listed in a table, including their age, body mass index, race, and previous treatments for OAB. No statistical analyzes were performed on the data, individual and grouped scores of the questionnaires were listed and presented as Tukey boxplots per timepoint. The answers to the questionnaires are listed as numbers and percentages. For the five specific TEAEs in the ePRO and QoL questionnaires, the sum of values was collected for each patient, and the means for the groups were calculated for each timepoint.

## Results

### Patient disposition

**Figure 4.**
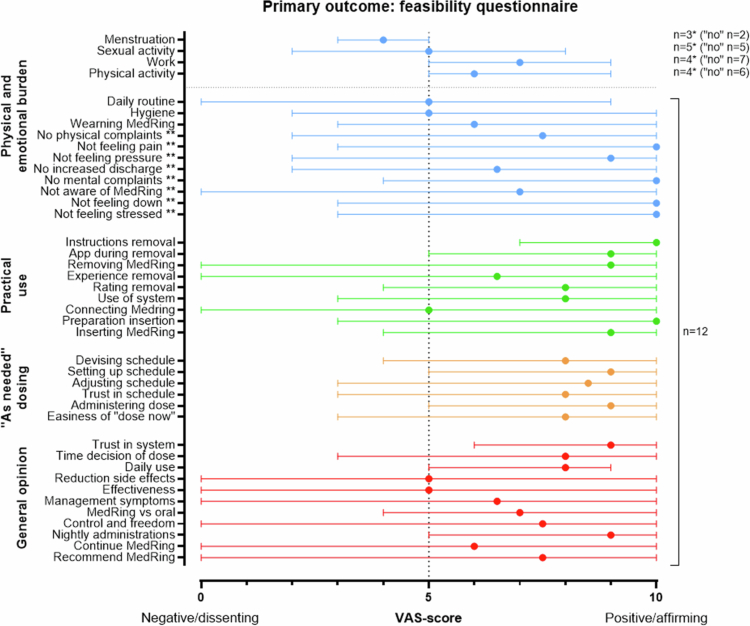
Primary outcome: feasibility questionnaire. *optional answers to these four questions were ‘yes,’ ‘no,’ and ‘not applicable.’ Only patients who answered ‘yes’ gave a score on the VAS scale. Patients who answered ‘no’ are added in brackets and the latter of patients answered ‘not applicable.’ **To present the data consistently, these questions were inverted and shown as a negatively phrased question. The original questions are outlined in the Supplementary Questionnaire 1. Full list of questions is listed in the Supplementary Questionnaire 1.

The study was conducted from February 2024 to August 2024. After screening, 13 patients were included, 10 of which 10 patients finalized the third period. Three patients discontinued the study due to different reasons (Figure 4). Patient 11 discontinued participation after 14 days and was replaced by a new patient, as the reason for discontinuation was unrelated to the study intervention.

Patient characteristics and demographics are summarized in Table 1, which shows that postmenopausal women had a higher BMI of 28.5, compared to premenopausal women who had a BMI of 24.5. Moreover, more premenopausal women received prior treatments, while all postmenopausal women had at least pelvic physiotherapy in the past, compared to half of the premenopausal women.

**Table 1.** Patient demographics.

	Premenopausal	Postmenopausal	Total
Number (*N*)	8	5	13
Age (years)			
Mean (SD)	40.5 (7.9)	59.6 (4.8)	47.8 (11.7)
Min, Max	31, 52	53, 64	31, 64
BMI (kg/m^2^)			
Mean (SD)	24.4 (3.96)	28.5 (3.50)	26.0 (4.18)
Min, Max	21.1, 33.3	23.6, 33.3	21.1, 33.3
Race (*N* (%))			
Asian	1 (12.5%)	0 (0%)	1 (7.7%)
Black or African American	1 (12.5%)	0 (0%)	1 (7.7%)
White	6 (75.0%)	5 (100%)	11 (84.6%)
Previous medication (*N* (%))			
Oxybutynin	2 (25%)	1 (20%)	3 (23.1%)
Solifenacin	1 (12.5%)	0 (0%)	1 (7.7%)
Botox injection	1 (12.5%)	0 (0%)	1 (7.7%)
Other/not specified medication	1 (12.5%)	1 (20%)	2 (15.4%)
Other treatment (*N* (%))			
Pelvic physiotherapy	4 (50%)	5 (100%)	9 (69.2%)
No previous treatment	2 (25%)	0 (0%)	2 (15.4%)

### Treatment exposure

In total, 96% of the planned administrations were given. Missed administrations were caused by DDs (see below), or instructed or uninstructed removal of the MedRing. Three different patients were instructed by the investigator to remove the MedRing because of a DD on days 2 (*n* = 1), 8 (*n* = 1), and 24 (*n* = 1). In other occasions, patients took the initiative to remove the MedRing for the following reasons: to clean (*n* = 2), during a menstruation (*n* = 1), during intercourse (*n* = 1), and due to difficulty in voiding (*n* = 1).

### Feasibility

Answers to the MedRing OAB questionnaire (*n* = 12 patients) are outlined in Figure 3. This questionnaire did not include the outcomes of patient 5 who discontinued after 1 week.

Overall, patients reported little physical or emotional burden from the MedRing OAB system, with median VAS scores ranging from 0 to 6. For questions applicable only to specific situations (e.g. menstruation, sexual activity), most patients reported no burden or indicated that the question was not applicable. Among the minority who did experience burden in these situations, the burden was limited, with medians between 4 and 7 (Figure 3, questions marked with an asterisk).

Practical use of the MedRing OAB was rated a median of 10 for the preparation on insertion and a median of 9 for the insertion of the MedRing on the VAS slider, indicating that both instructions were considered clear. Although one patient was unable to remove the MedRing herself despite additional instructions, the removal process was generally reported as positive. However, synchronization between the smartphone application and the MedRing was rated with high variability with a median of 5 and a range from 0 to 10. All ‘as needed’ dosing questions for a personalized schedule were positively scored with a median of 8 or higher.

Finally, most patients expressed positive opinions regarding the MedRing OAB system (Figure 3). They reported high levels of trust in the system, felt it offered increased control and freedom over their treatment, and particularly valued the option of nightly administrations. While opinions on continued personal use varied, most patients indicated they would recommend the MedRing OAB system to others.

### Safety and tolerability

Via the ePRO application, 32 different TEAEs were reported in 11 patients in the open text fields (Supplementary Table 1). The most frequently reported TEAE was a dry mouth, which was reported in 7 patients. The mean sum of values of the 5 specifically asked TEAEs in ePRO increased slightly over time.

During the visits, a total of 49 TEAEs were reported, of which 47 TEAEs were mild, 1 was moderate and 1 severe. All TEAEs are outlined in Supplementary Table 2, including their relatedness to oxybutynin and MedRing. The single severe TEAE was an allergic reaction to peanuts 6 days after the last dose which was considered unrelated to the study intervention. The single moderate TEAE was postmenopausal vaginal blood loss of the atrophic vaginal wall, which was most likely caused by the speculum used during gynecological examination. The most common TEAEs reported during a study visit were dry mouth, fatigue, headachem and pelvic pain. Device-related AEs, which were unrelated to oxybutynin, were pelvic pain and changes in discharge, both reported in two patients. These pelvic pain AEs occurred while riding a bike and during intercourse.

There were no local AEs or abnormalities during gynecological inspection caused by the MedRing. While three postmenopausal patients experienced vaginal blood loss related to vaginal atrophy, all patients tolerated the MedRing well. There were no abnormalities in the other safety parameters.

### Functioning

Two software updates were performed during the first three patients' participation to improve the functioning of the MedRing OAB system. The first update was to align the dates of the smartphone application to the MedRing (Table 2, severe DD patient 2). In the second update, the data transfer during the synchronizations was successfully shortened by removal of the temperature data from each synchronization to a new offboarding step at the end of the study.

**Table 2.** Device deficiencies per MedRing, in chronological order of patients.

Patient*		Time used	Device deficiency	Grade	Consequence
1		23 days	4× synchronization problem	Mild	Four missed administrations
			For unknown cause, MedRing had turned off	Severe	Fifteen missed administrations
2		28 days	Difficulty turning on MedRing	Mild	Used magnet instead of flashlight to turn on
			Dosing occurred on a different day due to time difference between smartphone application and MedRing	Severe	Smartphone application update
			Synchronization problem	Mild	One missed administration
3	A	NA	MedRing could not be installed	Moderate	MedRing was replaced
	B	21 days	2× synchronization problem	Mild	Two missed administrations
4		28 days	Synchronization problem	Mild	Three missed administrations
			Offboarding failed	Mild	Temperature logs retrieved via external device
5		7 days	-		-
6		28 days	-		-
7	A	7 days	Could not synchronize after installation. MedRing was removed to confirm dosing.	Moderate	One missed administration. MedRing replaced on day 7
	B	21 days	Offboarding failed	Mild	Temperature logs retrieved via external device
8	A	NA	MedRing could not be installed	Moderate	MedRing was replaced
	B	28 days	Unknown cause	Mild	One missed administration
			Synchronization problem did not overrule dosing schedule	Mild	Different dosing schedule, no missed administration
			Offboarding failed	Mild	Temperature logs retrieved via external device
9	A	NA	MedRing could not be installed	Moderate	MedRing was replaced
	B	28 days	Synchronization problem	Mild	One missed administration
10		28 days	Scheduled administrations at 6:00 were not administered, because the days split at 6:00 in the smartphone application	Mild	Six missed administrations
			Synchronization problem	Mild	Fifteen missed administrations
11		14 days	-		-
12		28 days	-		-
13		28 days	Synchronization problem	Mild	One missed administration

* Patients numbered in chronological order of the first day of study treatment.

In total, 29 DDs were recorded, which included 24 DDs of the MedRings (Table 2) 4 DDs of the smartphone application (data not shown) and 1 DD of the MedRing log. The single DD of the MedRing log was severe: the MedRing log displayed two 2 mg doses twice in patient 3. Due to suspected overdose, patients 3 and 4 were instructed to remove the MedRing. Further investigation showed that the DD resulted from an error in the display in the MedRing log. Subsequently, patient 4 re-inserted the MedRing, but patient 3 was withdrawn from the study by the physician, because this incident was considered to have strongly affected her experience.

Although 1 MedRing had been turned off due to an unknown cause by the programmed safety software, the battery life of the MedRings was sufficient at the end of the study and MedRing logs of all 13 MedRings were retrieved. The MedRing logs showed that all patients had frequently synchronized the smartphone application with the MedRing. However, synchronization difficulties occurred at least once in all patients (Table 2). Due to a technical labeling error the data on the adjustments of the dosing schedules is not reported. However, the actual updates in the schedules were retrievable. In the second and third treatment period, the cumulative number of schedule changes was 219 times. In the third treatment period, 6 of the 12 patients changed one or more dose volumes to 1 mg. Complete temperature MedRing logs were retrieved in 12 of the 13 patients; 1 log was unretrievable due to technical difficulties. All temperature measurements below 35.5 Celsius degrees corresponded with the reported MedRing removals by the patients. Moreover, all patients opened the smartphone application multiple times a week and read the FAQ and instructions several times.

The postcalibration output test was successfully completed in 12 of the 14 MedRings used during the study. The result of 1 MedRing was considered unreliable, as the test was conducted 7 days after retrieval. For the MedRing with the unexpected battery shutdown, the test was not performed because it was not incorporated in the procedures. Additionally, air bubbles were observed emerging from the drug outlet during the postcalibration test in 5 MedRings. The developer of the MedRing initiated investigations parallel to the conduction of the study to identify the underlying cause of the air bubbles.

### Pharmacokinetics

**Figure 5.**
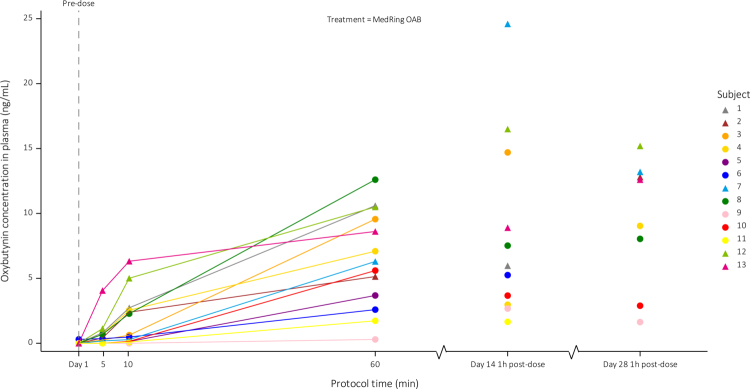
Individual oxybutynin measurements. Abbreviations: ng/mL = nanogram per milliliters, h = hour(s), m = minutes. Patients numbered in chronological order of the first day of study treatment. Subject six had measurable oxybutynin levels predose, despite discontinuation of oral oxybutynin according to protocol.

Individual plasma concentration‒time profiles are presented in Figure 5. This shows a rapid uptake of oxybutynin after administration at day 1. Measurable plasma concentrations were detected in 9 of 13 patients within 5 minutes postdose and in 12 of the 13 patients within 10 minutes postdose. At days 14 and 28 of treatment, all patients had measurable plasma levels of oxybutynin and DEOB.

### Satisfaction and quality of life

In total, 93.4% of the MedRing OAB questionnaires at days 1, 7, 14, 21, and 35 were completed. Responses to the identical questions of the primary outcome at days 14 and 35 did not differ significantly from those at day 28 (data not shown). The majority of the users were pleased to use the MedRing OAB and rated the smartphone application with a median of 7.5.

**Figure 6.**
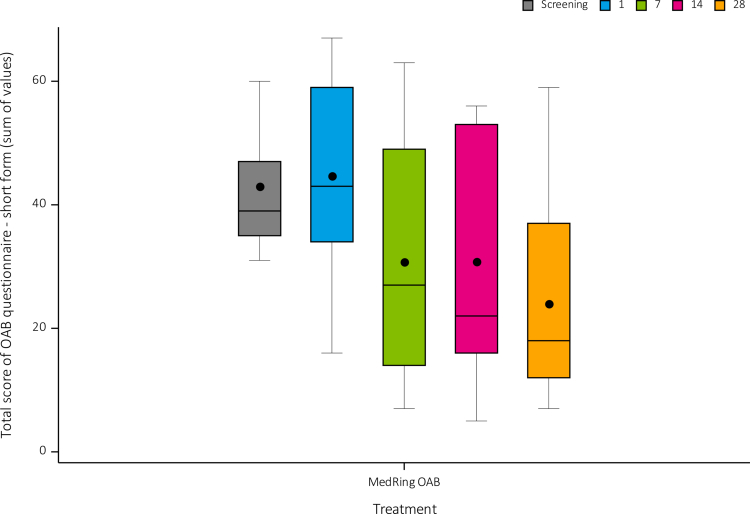
Sum of values for QoL questionnaire. Abbreviation: QoL = quality of life. Tukey boxplot: Black dot = Mean. This questionnaire had a minimum score of 0 (i.e. never any scenario applicable to their situation) and a maximum score of 95 (i.e. always all scenarios applicable to their situation). The mean sum of values (min-max) decreased from 44.7 (16 to 67) on day 1 (predose), to 30.8 (7 to 63) on day 7 and 30.8 (5 to 56) on day 14 and to 24.0 (7 to 59) on day 28.

For the QoL questionnaire, 96.2% of the questionnaires were completed. A decrease in the mean sum of values was seen over the course of the treatment period representing a reported improvement in QoL. The mean sum of values was decreased with 20 points between days 1 and 28 (Figure 6).

## Discussion

The primary aim of the present study was to assess the feasibility, tolerability, and safety of intravaginal oxybutynin administration in OAB patients via the MedRing OAB system for 28 days in an outpatient setting. Second, the functioning, user satisfaction and quality of life of OAB patients were assessed after 28 days. Our study was conducted in a small population of end-users, which allows feasibility assessment in the early stages of the clinical development of the MedRing OAB system. Finally, this was the first study to assess an intravaginal device operated by a smartphone application.

The present study shows that the MedRing OAB system is feasible, well tolerated and safe to use for 28 days in this population. Moreover, intravaginal dosing – including flexible, nightly, and on-demand dosing – resulted in rapid absorption and sufficient oxybutynin plasma levels over a 28-day period. Not surprisingly, for this first feasibility study, several challenges were encountered and remedied. During the study, 2 software updates were implemented in order to overcome technical limitations, resulting in an improvement in scheduling and optimized data transfer to enhance the functionalities of the MedRing OAB system.

The feasibility questionnaire showed that the MedRing OAB system was associated with little to no physical or emotional burden, was considered a practical system, had high scores for personalized schedules, which resulted in an overall positive opinion. It should be noted that the MedRing OAB questionnaire was specifically developed for the present study and not formally validated. Similar acceptability, satisfactory, and tolerability questions were used in other studies with intravaginal rings (Nel et al. 2021; Shapley-Quinn et al. 2023; Taruberekera et al. 2025). These reported equivalent outcomes for feasibility and usability as in our study. In general, intravaginal rings are reported as comfortable, with a high acceptability among users and feasible for intended treatment (Nel et al. 2021; Shapley-Quinn et al. 2023; Taruberekera et al. 2025). Our findings were also comparable to the findings in other studies with regard to the positive feedback on insertion and removal of the ring (Novák et al. 2003; Weinrib et al. 2020; Taruberekera et al. 2025). Similarly to our study, one of these studies specifically reported that a hormonal vaginal ring did not affect the sexual activities of the majority of the women (Novák et al. 2003). Taken together, our results showing the feasibility of MedRing OAB are in line with previous research on intravaginal rings.

Drug delivery by the MedRing OAB system never crossed the preset safety limits of the maximum dosing or time between administrations over the entire 28-day study period, which emphasizes the reliability of the safety mechanism of this system. The TEAEs considered related to oxybutynin were consistent with the side effect profile of intravaginal oxybutynin determined in the previous study with the MedRing OAB (Peltenburg et al. 2025, submitted). Additionally, the MedRing OAB system was well tolerated in all patients for 28 days, despite local TEAEs. Local AEs that were similar to our findings, such as a change in fluor or pelvic discomfort, have been reported in various pessary studies (Sarma et al. 2009; Bugge et al. 2020). Specifically, local TEAEs that were related to the device but unrelated to oxybutynin were comparable to device-related TEAEs observed in a 28-day study involving a vaginal ring for HPV prevention (Nel et al. 2016; Nel et al. 2021). Based on such previous experiences with long-term use of intravaginal devices, the burden of these mild local device-related symptoms is usually limited. In addition, our patients seem to be willing to tolerate mild local symptoms if they experience improvement of the OAB symptomatology.

The MedRing system functioned well throughout the study period of 28 days. Although synchronization difficulties occurred in each patient, the two software updates and an assumed learning curve resulted in a reduction of the occurrence of DDs. Moreover, temperature measurements caught all reported removals of the MedRing OAB and can therefore be used for treatment adherence as was previously described for the DST nano-T device (Boyd et al. 2015). MedRing logs showed that all patients used the smartphone application to adjust the schedule to their needs. Therefore, in this population, the variety in the use of the MedRing OAB system highlights the desire for personalized patient care.

Total administrations of oxybutynin showed a 96% success rate and sufficient oxybutynin plasma levels were measured after 14 and 28 days, indicating a successful administration of oxybutynin via the MedRing OAB system for 28 days. However, air bubbles were observed during the postcalibration output test, causing uncertainty about the presence of air during intravaginal dosing. Therefore, parallel investigations have been initiated to assess this unexpected finding.

Limitations of the present study are inherent to the exploratory, open-label and adaptive study design in a small population. Generalizing these findings to a broader population is therefore not justified, although we have attempted to reduce recall bias by regularly sending the MedRing OAB questionnaires to the patients and actively asking for TEAEs during visits. We consider the results of this study to be a starting point for future quantitative studies in OAB patients as it provides a framework for advancing the MedRing OAB system towards an intravaginal administration device focused on personalized patient care.

In conclusion, the MedRing OAB system appears to be a feasible, tolerable, and safe alternative for adequate, personalized administration of oxybutynin in an outpatient setting for 28 days in OAB patients. Further development of the MedRing OAB system will require additional clinical data and integration of patient feedback to optimize the product for future use.

## Supplementary Material

Supplementary material CHDR2222_Supplementary_25Sept2025_SPeltenburg.docx

Supplementary material CHDR2222_CONSORT checklist_23Oct2025.pdf

Supplementary material CHDR2222_CONSORT flowchart_23Oct2025.doc

## Data Availability

Data are available for bona fide researchers, who request it from the authors.
