# Supplementary material for: Introducing personalized patient care in overactive bladder management using the MedRing OAB system for intravaginal oxybutynin administration
Source: Drug Deliv. 2026 Jan 24;33(1):2617683. doi: 10.1080/10717544.2026.2617683 (PMC12833890; doi:10.1080/10717544.2026.2617683)
Supplement: Supplementary material — CHDR2222_Supplementary_25Sept2025_SPeltenburg.docx [file IDRD_A_2617683_SM1160.docx]

Supplementary

**Supplementary questionnaire 1: questions in MedRing OAB questionnaires**

**Day 1 Pre-installation MedRing smartphone app**

1. Do you experience side effects from the medication? Yes, open text/No
2. Do you experience a dry mouth? (0 = not at all / 10 = very much)
3. Do you experience dry eyes? (0 = not at all / 10 = very much)
4. Do you experience constipation? (0 = not at all / 10 = very much)
5. Do you experience reduced sweating? (0 = not at all / 10 = very much)
6. Do you experience dry skin? (0 = not at all / 10 = very much)
7. What is your first impression of the system? (0 = not good at all / 10 = excellent)
8. Design of the MedRing (0 = not good at all / 10 = excellent)

**Day 1 Pre-installation MedRing**

1. Support in the Companion app (0 = difficult / 10 = easy)
2. Preparation for insertion (0 = unclear / 10 = clear)

**Day 1 Post-installation MedRing**

1. Inserting the MedRing was (0 = difficult / 10 = easy)
2. Removing the MedRing was (0 = difficult / 10 = easy)
3. Activating the app was (0 = difficult / 10 = easy)
4. Overall rating of the installation process (0-10)
5. Connecting the app to the MedRing was (0 = difficult / 10 = easy)
6. Posture during insertion (laying down, squat-position, one leg up, other)

**Day 2**

1. Do you experience side effects from the medication? Yes, open text/No
2. Do you experience a dry mouth? (0 = not at all / 10 = very much)
3. Do you experience dry eyes? (0 = not at all / 10 = very much)
4. Do you experience constipation? (0 = not at all / 10 = very much)
5. Do you experience reduced sweating? (0 = not at all / 10 = very much)
6. Do you experience dry skin? (0 = not at all / 10 = very much)
7. Number of times I had to urinate today (< 5 times, 5-7 times, 7-10 times, >10 times)
8. The volume of each void was (little/average/a lot)
9. Number of times I had unwanted urine loss (incontinence)

**Day 7**

1. Do you experience side effects from the medication? Yes, open text/No
2. Do you experience a dry mouth? (0 = not at all / 10 = very much)
3. Do you experience dry eyes? (0 = not at all / 10 = very much)
4. Do you experience constipation? (0 = not at all / 10 = very much)
5. Do you experience reduced sweating? (0 = not at all / 10 = very much)
6. Do you experience dry skin? (0 = not at all / 10 = very much)
7. Number of times I had to urinate today (< 5 times, 5-7 times, 7-10 times, >10 times)
8. The volume of each void was (little/average/a lot)
9. Number of times I had unwanted urine loss (incontinence)
10. How would you rate the ease of use of the void diary? (0 = difficult / 10 = easy)

**Day 14**

1. Do you experience side effects from the medication? Yes, open text/No
2. Do you experience a dry mouth? (0 = not at all / 10 = very much)
3. Do you experience dry eyes? (0 = not at all / 10 = very much)
4. Do you experience constipation? (0 = not at all / 10 = very much)
5. Do you experience reduced sweating? (0 = not at all / 10 = very much)
6. Do you experience dry skin? (0 = not at all / 10 = very much)
7. Number of times I had to urinate today (< 5 times, 5-7 times, 7-10 times, >10 times)
8. The volume of each void was (little/average/a lot)
9. Number of times I had unwanted urine loss (incontinence)
10. Did the MedRing affect your menstruation and to what extent (not applicable, no, 0 = very negative / 10 = very positive)
11. Did the MedRing affect your sexual activity and to what extent (not applicable, no, 0 = very negative / 10 = very positive)
12. To what extent does the MedRing affect your daily routine (0 = very negative / 10 = very positive)
13. Has the MedRing affected your work and work-related activities and to what extent? (not applicable, no, 0 = very negative / 10 = very positive)
14. Has the MedRing affected your physical activities and to what extent? (not applicable, no, 0 = very negative / 10 = very positive)
15. To what extent does the MedRing affect your personal hygiene? (0 = very negative / 10 = very positive)

**Dag 21**

1. Do you experience side effects from the medication? Yes, open text/No
2. Do you experience a dry mouth? (0 = not at all / 10 = very much)
3. Do you experience dry eyes? (0 = not at all / 10 = very much)
4. Do you experience constipation? (0 = not at all / 10 = very much)
5. Do you experience reduced sweating? (0 = not at all / 10 = very much)
6. Do you experience dry skin? (0 = not at all / 10 = very much)
7. Number of times I had to urinate today (< 5 times, 5-7 times, 7-10 times, >10 times)
8. The volume of each void was (little/average/a lot)
9. Number of times I had unwanted urine loss (incontinence)
10. How do you experience the ease of use of the insights in the Companion App? (0 = difficult / 10 = easy)

**Dag 28**

1. Do you experience side effects from the medication? Yes, open text/No
2. Do you experience a dry mouth? (0 = not at all / 10 = very much)
3. Do you experience dry eyes? (0 = not at all / 10 = very much)
4. Do you experience constipation? (0 = not at all / 10 = very much)
5. Do you experience reduced sweating? (0 = not at all / 10 = very much)
6. Do you experience dry skin? (0 = not at all / 10 = very much)
7. Number of times I had to urinate today (< 5 times, 5-7 times, 7-10 times, >10 times)
8. The volume of each void was (little/average/a lot)
9. Number of times I had unwanted urine loss (incontinence)
10. Have you experienced any physical complaints from the MedRing? (0 = none / 10 = a lot)
11. Have you experienced pain at the location of the MedRing? (0 = none / 10 = a lot)
12. Have you experienced pressure at the location of the MedRing? (0 = none / 10 = a lot)
13. Have you experienced increased vaginal discharge while wearing the MedRing? (0 = much less / 10 = much more)
14. Have you experienced mental complaints from the MedRing system? (0 = none / 10 = a lot)
15. To what extent were you aware of the presence of the MedRing? (0 = not at all / 10 = very much)
16. How did you feel about wearing the MedRing continuously? (0 = unpleasant / 10 = pleasant)
17. Have you felt more down in the past days than before the MedRing installation? (0 = not at all / 10 = completely)
18. Have you felt more stress in the past days than before the MedRing installation? (0 = not at all / 10 = completely)
19. Did the MedRing affect your menstruation and to what extent (not applicable, no, 0 = very negative / 10 = very positive)
20. Did the MedRing affect your sexual activity and to what extent (not applicable, no, 0 = very negative / 10 = very positive)
21. To what extent does the MedRing affect your daily routine (0 = very negative / 10 = very positive)
22. Has the MedRing affected your work and work-related activities and to what extent? (not applicable, no, 0 = very negative / 10 = very positive)
23. Has the MedRing affected your physical activities and to what extent? (not applicable, no, 0 = very negative / 10 = very positive)
24. To what extent does the MedRing affect your personal hygiene? (0 = very negative / 10 = very positive)
25. Removing the MedRing was (0 = difficult / 10 = easy)
26. How did you experience removing the MedRing? (0 = unpleasant / 10 = very pleasant)
27. Clarity of instructions for removal (0 = unclear / 10 = completely clear)
28. Use of the app during MedRing removal (0 = difficult / 10 = easy)
29. Overall rating of the removal process (0-10)
30. Using the MedRing system was (0 = unpleasant / 10 = pleasant)
31. Connecting to the app was (0 = difficult / 10 = easy)
32. Adjusting a medication release schedule was (0 = difficult / 10 = easy)
33. Ease of installing personal schedule in app (0 = difficult / 10 = easy)
34. Ease of devising personal schedule (0 = difficult / 10 = easy)
35. Trust in personal schedule (0 = little / 10 = a lot)
36. Ease of use of administering next dose now (0 = difficult / 10 = easy)
37. Administering a dose was (0 = difficult / 10 = easy)
38. I had trust in the system (0 = little / 10 = a lot)
39. Rating of daily use of MedRing system (0-10)
40. I would recommend the MedRing to other patients (0 = not true / 10 = true)
41. I would like to continue using the MedRing system (0 = not true / 10 = true)
42. The MedRing is more pleasant than taking medication orally (0 = not true / 10 = true)
43. I experienced fewer side effects of this medication using the MedRing (0 = not true / 10 = true)
44. The medication was more effective with the MedRing compared to my usual method of use (0 = not true / 10 = true)
45. Decision on the timing of dosing (0 = difficult / 10 = easy)
46. Using the MedRing gives me more control and freedom (0 = not true / 10 = true)
47. The MedRing helps me to manage my overactive bladder symptoms better (0 = not true / 10 = true)
48. It is an advantage that the MedRing can administer medication at night (0 = not true / 10 = true)

**Day 35**

1. Have you experienced physical complaints from the MedRing? (0 = little / 10 = a lot)
2. Have you experienced mental complaints from the MedRing system? (0 = little / 10 = a lot)
3. Did the MedRing affect your menstruation and to what extent (not applicable, no, 0 = very negative / 10 = very positive)
4. Did the MedRing affect your sexual activity and to what extent (not applicable, no, 0 = very negative / 10 = very positive)
5. To what extent does the MedRing affect your daily routine (0 = very negative / 10 = very positive)
6. Has the MedRing affected your work and work-related activities and to what extent? (not applicable, no, 0 = very negative / 10 = very positive)
7. Has the MedRing affected your physical activities and to what extent? (not applicable, no, 0 = very negative / 10 = very positive)
8. To what extent does the MedRing affect your personal hygiene? (0 = very negative / 10 = very positive)
9. Using the MedRing and Companion App was (0 = unpleasant / 10 = pleasant)
10. Connecting to the Companion App was (0 = instable / 10 = stable)
11. I had trust in the system (0 = little / 10 = a lot)
12. Administering a dose was (0 = difficult / 10 = easy)
13. Adjusting a medication release schedule was (0 = difficult / 10 = easy)
14. I would recommend the MedRing to other patients (0 = not true / 10 = true)
15. I would like to continue using the MedRing system (0 = not true / 10 = true)
16. Rating of daily use of MedRing system (0-10)
17. The MedRing is more pleasant than taking medication orally (0 = not true / 10 = true)
18. I experienced fewer side effects of this medication using the MedRing (0 = not true / 10 = true)
19. The medication was more effective with the MedRing compared to my usual method of use (0 = not true / 10 = true)
20. Decision on the timing of dosing (0 = difficult / 10 = easy)
21. 3 advantages (open)
22. 3 disadvantages (open)
23. Number of times I had to urinate today (< 5 times, 5-7 times, 7-10 times, >10 times)
24. The volume of each void was (little/average/a lot)
25. Number of times I had unwanted urine loss (incontinence)

**Supplementary table 1: AEs reported in ePRO (translated)**

| System organ class/  preferred term | Study day | | | | | Total | |
| --- | --- | --- | --- | --- | --- | --- | --- |
|  | **Day 2** Events | **Day 7** Events | **Day 14** Events | **Day 21** Events | **Day 28** Events |  |  |
|  |  |  |  |  |  | Events | N |
| ANY EVENTS | **8** | **12** | **12** | **16** | **16** | **64** | **11** |
| GASTROINTESTINAL DISORDERS | 2 | 6 | 7 | 11 | 8 |  |  |
| Abdominal pain | - | 1 | 1 | - | - | 2 | 1 |
| Dry mouth | 1 | 2 | 1 | 6 | 3 | 13 | 7 |
| Dry throat | - | 1 | 1 | 1 | 1 | 4 | 1 |
| Thirst | - | - | 1 | - | - | 1 | 1 |
| Gastroesophageal reflux | - | - | 1 | 2 | 2 | 5 | 2 |
| Dysphagia | - | - | 1 | 1 | - | 2 | 1 |
| Nausea | 1 | 1 | 1 | 1 | 1 | 5 | 2 |
| Vomiting | - | 1 | - | - | - | 1 | 1 |
| Loose stool | - | - | - | - | 1 | 1 | 1 |
| GENERAL DISORDERS | - | 1 | 1 | 1 | 1 |  |  |
| Somnolence | - | 1 | 1 | 1 | 1 | 4 | 1 |
| RESPIRATORY DISORDERS | - | - | - | - | 1 |  |  |
| Cough | - | - | - | - | 1 | 1 | 1 |
| MUSCULOSKELETAL DISORDERS | - | 1 | 1 | - | - |  |  |
| Back pain | - | 1 | 1 | - | - | 2 | 1 |
| NERVOUS SYSTEM DISORDERS | 2 | 2 | - | 1 | 1 |  |  |
| Headache | 2 | 2 | - | 1 | 1 | 6 | 2 |
| REPRODUCTIVE DISORDERS | 4 | 1 | 1 | 1 | 2 |  |  |
| Pelvic pain | 2 | 1 | 1 | - | 1 | 5 | 2 |
| Vaginal discomfort | 1 | - | - | 1 | 1 | 3 | 1 |
| Vaginal laceration | 1 | - | - | - | - | 1 | 1 |
| SKIN DISORDERS | - | 1 | 2 | 2 | 3 |  |  |
| Acne | - | - | 1 | - | - | 1 | 1 |
| Dry skin | - | - | 1 | 1 | 2 | 4 | 2 |
| Oedema | - | 1 | - | 1 | - | 2 | 2 |
| Scalp pruritis | - | - | - | - | 1 | 1 | 1 |

**Supplementary table 2: AEs reported during a visit**

| **System organ class/  preferred term** | **Oxybutynin related** | | | | | | **MedRing related** | | | | | |
| --- | --- | --- | --- | --- | --- | --- | --- | --- | --- | --- | --- | --- |
|  | **Probable** | | **Possible** | | **Unrelated** | | **Probable** | | **Possible** | | **Unrelated** | |
|  | Events | N | Events | N | Events | N | Events | N | Events | N | Events | N |
| ANY EVENTS (N = 49) | 8 | 4 | 24 | 11 | 17 | 10 | 7 | 5 | 11 | 6 | 31 | 12 |
| GASTROINTESTINAL DISORDERS | 7 | 4 | 3 | 3 | 2 | 2 | 1 | 1 | 1 | 1 | 10 | 5 |
| Abdominal pain | - | - | 1 | 1 | - | - | - | - | 1 | 1 | - | - |
| Abdominal pain upper | - | - | 1 | 1 | - | - | 1 | 1 | - | - | - | - |
| Diarrhea | - | - | 1 | 1 | - | - | - | - | - | - | 1 | 1 |
| Dry mouth | 4 | 4 | - | - | - | - | - | - | - | - | 4 | 4 |
| Dyspepsia | - | - | - | - | 1 | 1 | - | - | - | - | 1 | 1 |
| Gastroesophageal reflux disease | - | - | - | - | 1 | 1 | - | - | - | - | 1 | 1 |
| Nausea | 1 | 1 | - | - | - | - | - | - | - | - | 1 | 1 |
| Vomiting | 2 | 2 | - | - | - | - | - | - | - | - | 2 | 2 |
| GENERAL DISORDERS | - | - | 3 | 3 | - | - | - | - | - | - | 3 | 3 |
| Fatigue | - | - | 3 | 3 | - | - | - | - | - | - | 3 | 3 |
| IMMUNE SYSTEM DISORDERS | - | - | - | - | 1 | 1 | - | - | - | - | 1 | 1 |
| Food allergy | - | - | - | - | 1 | 1 | - | - | - | - | 1 | 1 |
| INFECTIONS AND INFESTATIONS | - | - | - | - | 4 | 3 | - | - | - | - | 4 | 3 |
| Ear infection bacterial | - | - | - | - | 1 | 1 | - | - | - | - | 1 | 1 |
| Respiratory tract infection | - | - | - | - | 2 | 2 | - | - | - | - | 2 | 2 |
| Urinary tract infection | - | - | - | - | 1 | 1 | - | - | - | - | 1 | 1 |
| INJURY COMPLICATIONS | - | - | 1 | 1 | - | - | - | - | - | - | 1 | 1 |
| Vulvovaginal injury | - | - | 1 | 1 | - | - | - | - | - | - | 1 | 1 |
| MUSCULOSKELETAL DISORDERS | - | - | 1 | 1 | 1 | 1 | - | - | 1 | 1 | 1 | 1 |
| Back pain | - | - | 1 | 1 | - | - | - | - | 1 | 1 | - | - |
| Pain in extremity | - | - | - | - | 1 | 1 | - | - | - | - | 1 | 1 |
| NERVOUS SYSTEM DISORDERS | - | - | 5 | 4 | - | - | - | - | - | - | 5 | 4 |
| Cognitive disorder | - | - | 2 | 1 | - | - | - | - | - | - | 2 | 1 |
| Headache | - | - | 3 | 3 | - | - | - | - | - | - | 3 | 3 |
| RENAL AND URINARY DISORDERS | 1 | 1 | 1 | 1 | 2 | 2 | 1 | 1 | 1 | 1 | 2 | 2 |
| Hypertonic bladder | 1 | 1 | 1 | 1 | 1 | 1 | - | - | 1 | 1 | 2 | 2 |
| Urinary retention | - | - | - | - | 1 | 1 | 1 | 1 | - | - | - | - |
| REPRODUCTIVE DISORDERS | - | - | 7 | 5 | 6 | 5 | 5 | 4 | 8 | 5 | - | - |
| Dysmenorrhea | - | - | 1 | 1 | 1 | 1 | - | - | 2 | 2 | - | - |
| Genital discharge | - | - | - | - | 2 | 2 | 2 | 2 | - | - | - | - |
| Heavy menstrual bleeding | - | - | 1 | 1 | - | - | - | - | 1 | 1 | - | - |
| Intermenstrual bleeding | - | - | 1 | 1 | - | - | - | - | 1 | 1 | - | - |
| Menstruation irregular | - | - | 1 | 1 | - | - | - | - | 1 | 1 | - | - |
| Pelvic pain | - | - | 1 | 1 | 2 | 2 | 2 | 2 | 1 | 1 | - | - |
| Vaginal discharge | - | - | - | - | 1 | 1 | 1 | 1 | - | - | - | - |
| Vaginal hemorrhage | - | - | 1 | 1 | - | - | - | - | 1 | 1 | - | - |
| Vulvovaginal discomfort | - | - | 1 | 1 | - | - | - | - | 1 | 1 | - | - |
| SKIN DISORDERS | - | - | 3 | 3 | 1 | 1 | - | - | - | - | 4 | 4 |
| Acne | - | - | 1 | 1 | - | - | - | - | - | - | 1 | 1 |
| Dry skin | - | - | 1 | 1 | 1 | 1 | - | - | - | - | 2 | 2 |
| Erythema | - | - | 1 | 1 | - | - | - | - | - | - | 1 | 1 |
