# Supplementary material for: Introducing personalized patient care in overactive bladder management using the MedRing OAB system for intravaginal oxybutynin administration
Source: Drug Deliv. 2026 Jan 24;33(1):2617683. doi: 10.1080/10717544.2026.2617683 (PMC12833890; doi:10.1080/10717544.2026.2617683)
Supplement: Supplementary material — CHDR2222_CONSORT flowchart_23Oct2025.doc [file IDRD_A_2617683_SM1158.doc]

**CONSORT 2010 Flow Diagram**

**Allocation**

**Analysis**

**Follow-Up**

**Enrollment**

Assessed for eligibility (n=24)

Excluded (n=11)

♦  Not meeting inclusion criteria (n=11)

Analysed (n=13)
♦ Excluded from feasibility endpoint analysis (n=1)

- Subject discontinued study at day 7

Lost to follow-up (give reasons) (n=0)

Discontinued intervention (n=3)

- 1 withdrew consent on day 7 due to perimenopausal changes
- 1 withdrew consent on day 14 due the commuting time (unrelated to the study intervention)*
- 1 discontinued on day 21 due to suspected overdose, which was refuted

* this subject was replaced

Allocated to intervention (n=13)

♦ Received allocated intervention (n=13)

♦ Did not receive allocated intervention (give reasons) (n=0)

Lost to follow-up (give reasons) (n= )

Discontinued intervention (give reasons) (n= )

Allocated to intervention (n= )

♦ Received allocated intervention (n= )

♦ Did not receive allocated intervention (give reasons) (n= )

Analysed (n= )
♦ Excluded from analysis (give reasons) (n= )

Included (n=13)
